# Morten Møller: In Memoriam

**DOI:** 10.1111/jpi.70074

**Published:** 2025-09-22

**Authors:** Horst‐Werner Korf, Martin Fredensborg Rath

**Affiliations:** ^1^ Department Anatomy I Heinrich Heine University Duesseldorf Duesseldorf Germany; ^2^ Department of Neuroscience University of Copenhagen Denmark

**Keywords:** arcuate nucleus, electron microscopy, junctional complexes, pineal innervation, pineal organ

Morten Møller, Professor emeritus in Neuroanatomy, Department of Neuroscience, Faculty of Health Sciences, University of Copenhagen passed away on July 16, 2025. This sadly marks the end of a most remarkable scientific career which spanned six decades. Many friends and colleagues worldwide mourn his death (Figure 1).

**Figure 1:**
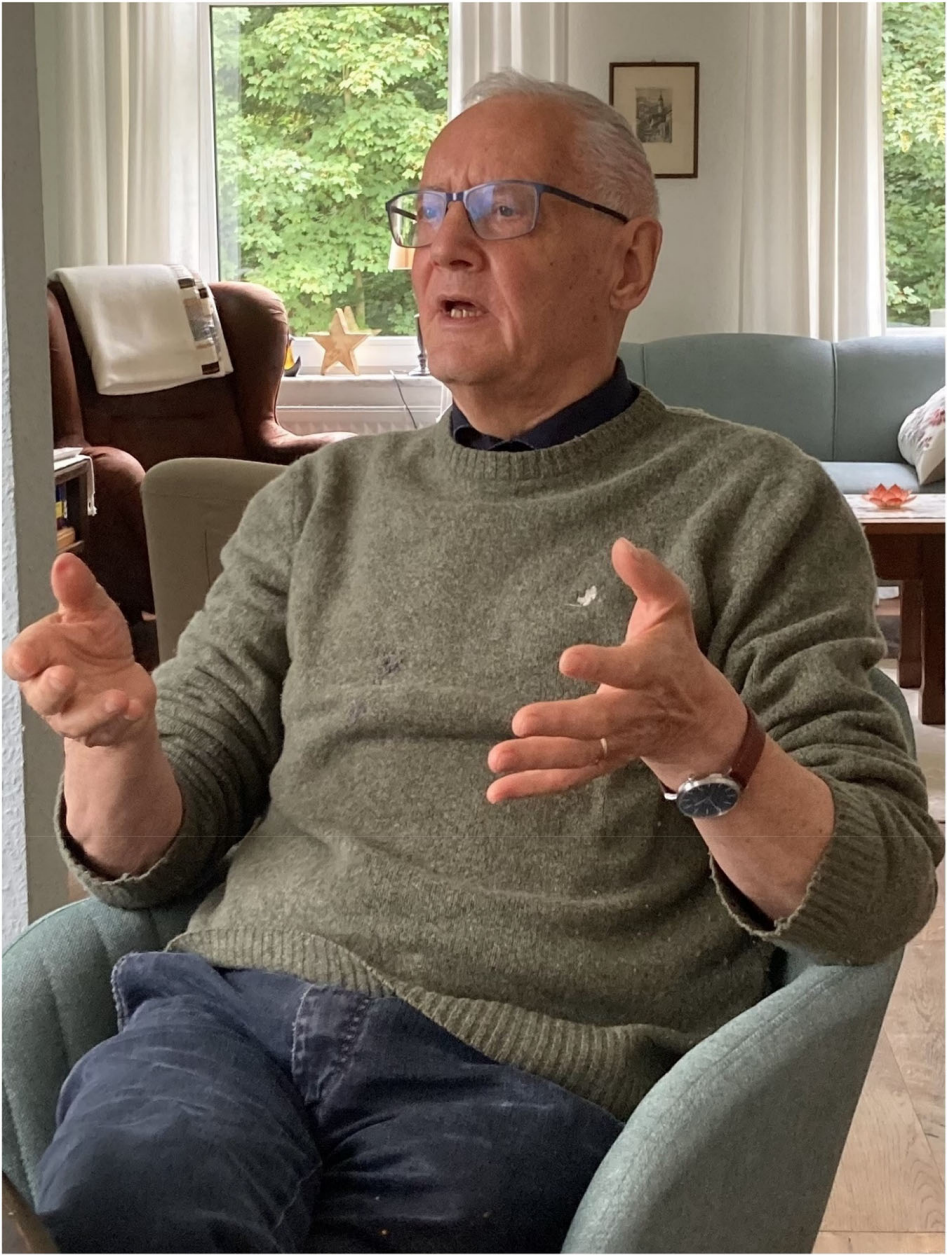
Morten Møller discussing his latest results on the ultrastructure of the rat pineal with Horst Korf in August 2023. Photograph taken by Beate Korf.

Morten Møller was born in Odense, Denmark on November 29, 1942. In 1969, Morten got married with Vera Gudjohnsen of Thyborøn, who accompanied and supported Morten for more than 50 years until she passed away on January 24, 2020. As Morten used to say “A secret in a man's life is his wife”. In the same year Morten graduated as MD from the University of Copenhagen and passed the American ECFMG‐examination. Thereafter, he worked as a medical intern in in Kansas City. In 1972, he joined the Institute of Medical Anatomy at the University of Copenhagen as research assistant and received tenure as an associate professor in 1976. In 1987, he defended his thesis as Dr. Med. Sci and was promoted full professor in Neuroanatomy in 1994. From 2001 to 2010, Morten served as director of the Graduate School of Neuroscience, University of Copenhagen, and thereafter he was appointed by the University of Copenhagen as Director of the Research Training Programme in Neuroscience and kept this office until 2014. On October 1, 2015, Morten retired and became Professor emeritus, but even after his retirement, Morten was actively engaged in research. On his very last day in the laboratory (May 30, 2024) he worked with the stereotactic frame and discussed recent electron microscopic data and grant applications, before he was hit by a severe stroke from which he unfortunately did not recover.

Morten Møller's research focused on the functional morphology of neuroendocrine systems in the mammalian brain. He loved to work in the laboratory and mastered multiple methods: electron microscopy, immunohistochemistry, tracing techniques, receptor autoradiography, and in‐situ hybridization. His initial studies investigated the pineal gland of human fetuses, in which he identified a central innervation establishing a direct connection between the pineal and the brain [1]. In those days, the concept that the mammalian pineal organ is solely innervated by postganglionic sympathetic nerve fibers had become a dominating dogma, but Morten's work has clearly shown that, in addition, the mammalian pineal organ is innervated by a plethora of axons originating from the brain as well as from parasympathetic and sensory ganglia. This diversified innervation, which also employs several neuropeptides and acetylcholine, has been a major theme of his research [2, 3, 4, 5, 6]. He has also contributed to studies demonstrating immunocytochemical similarities between retinal photoreceptors and pinealocytes that underpinned the concept of multiple types of mammalian pinealocytes [7, 8] whose functional differences have been further elaborated by Rath et al. [9]. In 2004, Morten started a very successful collaboration with David Klein at National Institutes of Health, Bethesda demonstrating expression of various genes in the rodent pineal gland, including homeobox gene‐encoded transcription factors, under the control of the noradrenergic‐cyclic AMP pathway and during development [9, 10, 11, 12, 13, 14, 15, 16, 17, 18, 19].

In his last years, Morten returned to ultrastructural investigations by employing serial block face scanning electron microscopy with high‐resolution 3‐dimensional analyses. This method allowed for identification of a “trans‐pineal tanycyte‐like cell” that connects the ventricular system with the subarachnoid space [20] and a novel junctional complex separating pinealocyte bulbous projections from their perikarya (Figure 2), which probably participate in paracrine glutamatergic inhibition of the melatonin secretion [21].

**Figure 2:**
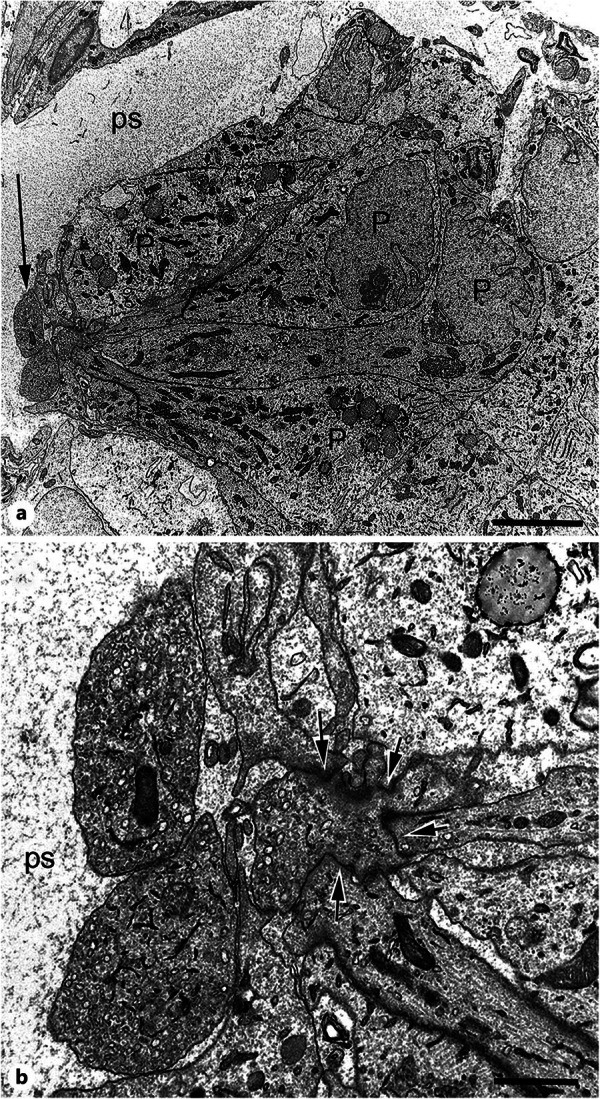
TEM micrographs showing bulbous processes from pinealocytes located in the perivascular space. a Pinealocytes with ovoid cell bodies (P) terminate with bulbous processes (arrow) directly in the perivascular space (ps). Scale bar = 5 µm. b High magnification of the bulbous processes showing the junctional complexes at the neck‐like origin of the process (arrows). ps, perivascular space. Scale bar = 1 µm. Reproduced from [21] with permission.

Morten also contributed investigations on hypothalamic neuroendocrine structures such as the vasopressinergic and oxytocinergic systems [22] and the arcuate‐median eminence complex [23], and on the blood‐brain barrier [24].

Morten Møller was a gifted academic mentor who trained several young scientists from all over the world in functional morphology: Jens Mikkelsen, Martin Fredensborg Rath, Anders‐Fink Jensen, Lone Helboe, Philip Just‐Larsen, Niels Vrang, Florian Baeres, Karen Bonde Larsen, Louise Rovsing, Denmark; Bruno Cozzi, Chiara Fabris, Italy; Valerie Simonneaux, France; Pansiri Phansuwan‐Pujito, Sujira Mukda, Piyarat Govitrapong, Thailand; Corian Badiu, Romania; James Olcese, USA; Liu Wei, China: Ana Coto‐Montes, Spain, most of whom became independent researchers or institute directors during later stages of their careers. Morten was a visiting fellow at Universities of Göttingen, Giessen, Mainz, and Frankfurt a.M., Germany.

Professor Møller was also an excellent teacher for medical students with high didactic skills. Even at the end of his career in 2023, he received outstanding evaluations from the students. Morten authored Danish textbooks in neuronanatomy with focus on excellent didactics combined with beautiful sections and preparations of the human brain. *Centralnervesystemets Anatomi* (Rath and Møller, 2020) has now become a classic in the medical education in Denmark.

In recognition of his scientific and academic achievements Morten Møller has been awarded with the “Ulrich and Maria Brinch's Scientific Honor Price” for studies of rhythm‐generating centers in the diencephalon (1990) and with the Neurocluster award for collaboration between basic and clinical sciences (2005). In 1995, he was elected as Honorary Member of the Romanian Academy of Sciences.

In addition to his predominant activities in research and teaching, Morten served the Danish Society for Neuroscience as founding member and chairman from 1992 to 1995 and as Danish representative in the International Brain Research Organization (IBRO) from 1993 to 1994 and in the European Cooperation in Science and Technology (COST) from 1996 to 2002. Morten also organized several Scandinavian and Danish meetings, inter alia: Scandem‐84 (Copenhagen, 1984), Sandbjerg Symposia (Sønderborg, 1987, 1988, 1995, 2004 and 2008), Denmark; 4th International Symposium on VIP, PACAP, Glucagon and related Peptides (Elsinore, 1999); 18th Meeting of the Scandinavian Sleep Research Society (Copenhagen, 2003), and Symposium on circadian gene expression in the brain (Copenhagen, 2004).

Of note, Morten has been a driving force for the formation and propagation of a European platform for pineal and biological rhythm research. He became a founding member of the European Pineal Study group (EPSG) and actively participated in all EPSG conferences (Amsterdam, 1978; Giessen, 1981; Pecs, 1984; Modena, 1987; Guildford, 1990). Because the membership base increased rapidly, the EPSG was transformed into the European Pineal Society (EPS) in 1990, upon suggestion by Jo Arendt, Guildford, and Morten organized the following congress of the society in Copenhagen (1993), a highlight with regard to science, but rather bad weather. In 1996, Morten was elected as President of the EPS and kept this position until 2002. As highly influential member of the executive board, Morten strongly supported the transformation of the European Pineal Study group/Society into the European Biological Rhythms Society (EBRS) executed in 2005 during the Congress in Frankfurt am Main to attract members working on organisms without a pineal organ (unicellular organisms, invertebrates, plants).

Morten took great care about people around him. In his younger years, he worked as an emergency doctor during nights and weekends and often talked about his calls to patients in all social classes, from ambassador residences to Christiania. Morten was a master in turning scientific collaboration into cordial friendship. Together with his wife, Vera, he offered warm hospitality to collaborators and their families in his red‐brick house and beautiful garden with excellent food and wine. He had a fantastic sense of humour and a very special temper. Thus, discussions and conversations, be they on a scientific or a personal level, were always vivid, sometimes he abruptly changed from communications in English to Danish language. Morten was a gifted singer and while he would never miss an opportunity to delve into the hymns of his home country, he was also very fond of deutsche Volkslieder, e.g. “Die Loreley”, which he could sing much better than (most of) his German colleagues.

Morten Møller is survived by his children Peder, Ivan, Thorsten and Nina and their families to whom we extend our great sympathy.

Kære Morten, you will be missed and remembered. Rest in peace.

## Data Availability

The data that support the findings of this study are available from the corresponding author upon reasonable request.
